# Well-being, behavioral patterns and cycling crashes of different age groups in Latin America: Are aging adults the safest cyclists?

**DOI:** 10.1371/journal.pone.0221864

**Published:** 2019-08-28

**Authors:** Sergio A. Useche, Francisco Alonso, Jaime Sanmartin, Luis V. Montoro, Boris Cendales

**Affiliations:** 1 INTRAS (Research Institute on Traffic and Road Safety) - Faculty of Psychology, University of Valencia, Valencia, Spain; 2 Faculty of Economic and Administrative Sciences, El Bosque University, Bogotá, Colombia; Tongii University, CHINA

## Abstract

**Objectives:**

This study aimed at analyzing the cycling safety-related factors and the mental health indicators of elderly cyclists in comparison with other age groups.

**Methods:**

For this cross-sectional study, we analyzed the data of 911 bicyclists from two Latin American countries that have been experiencing a substantial growth of urban cycling during the last few years: Colombia and Argentina. Participants responded to an e-questionnaire on bicycling behaviors, mental health and cycling safety.

**Results:**

Aging adults reported lower rates of risky behaviors and traffic crashes (around .38 in five years), and, on the other hand, more cycling protective behaviors, a higher risk perception and a better knowledge of traffic norms than both other adults (26–50 years old) and young cyclists (<26). Cycling behaviors and crashes were found to be significantly related to mental health indicators, the latter being higher in aging cyclists. However, this population remains more prone to distractions experienced while cycling than other age groups.

**Conclusions:**

Although the behavioral features of aging adults were comparatively “safer” than the ones displayed by other age groups, factors such as cycling distractions and this population’s over-representation in traffic crashes suggest the need of enforcing policymaking for a better integration of this age segment in alternative transportation dynamics.

## Introduction

As transportation dynamics are constantly changing, shifting in most countries to alternative and sustainable means of transport, different users are systematically replacing the use of traditional cars with non-motorized vehicles; among them, the bicycle particularly stands out, being often chosen by users for their urban trips, thanks to some of its key factors such as efficiency, health and environmental sustainability [1–3].

As stated in many recent studies, using active transport means for (e.g.), daily commuting, leisure and regular trips improves the individual’s general physical and mental health [4–6]. Different positive physical benefits, such as a decreased risk of suffering Type 2 –diabetes, cancer and cardiovascular diseases [7,8], and mental health outcomes, such stress reduction and subjective well-being [9–10], have been found in active urban cyclists, especially when compared to motor-vehicle commuters [7].

These benefits result specially interesting when applied to populations that are more prone to present numerous health problems and sedentary lifestyles, such as adults over 50 years old. In brief, promoting alternative transport means contributes to improve this population’s general health, and to prevent several issues that may affect them by means of physical exercise [11,12]. Enforced by policymaking, cycling has gained a significant popularity among population groups such as aging adults, who frequently perceive urban cycling as the instrument to achieve different improvements in physical health, lifestyle, autonomy and mental health [4,12].

### What factors can be responsible of putting aging cyclists at risk in Latin America?

Although cycling and other types of active commuting imply many proven health advantages, the epidemiological figures of the last decade have systematically shown how not everything is as good as it seems: in fact, healthy transportation may imply substantial risks for its users. This is especially true when we consider that cycling in Latin America has recently been growing very fast in a span of only a few years. In this regard, the *boom of cycling* in large Latin American cities is noticeable when we look at some brief facts: in Bogotá (Colombia), ranked in the *Copenhagenize Index* as the 12^th^ bike-friendliest city in 2019 (when it had never appeared in the ranking before) [13], more than 5% of all the urban journeys have been made by bike since 2015, and more than 580 km of exclusive bicycle lanes -integrated within other transport systems and daily used by more than two million people- are expected to be built by the end of 2019 [14]. In Buenos Aires (Argentina), more than 180,000 people use bikes as their main transport means on a daily basis, or utilize them to complement their trips otherwise carried out exclusively with public transport, thus having more than 200 bike stations and 230 km of bike lanes [15]. Also, Buenos Aires is ranked as the 14^th^ bike-friendliest urban center in the world for the year 2015 [16]. However, different concerns, barriers and challenges are still visible and stand against the safety of cyclists. In other words, the aforementioned key developments (infrastructure, integration, education and culture), although relevant, have not taken place at the same pace as the rapid spread of the use of bicycles for urban journeys [17–20].

Within the most concerning highlights provided by statistical reports, it is possible to see how elderly users constitute one of the population segments registering the most injuries and fatalities as cyclists [4,21], many of them enhanced by circumstances and patterns commonly observable in the Latin American context, such as the disuse of passive safety elements (for instance, helmets and reflective wearable features), and the lack of safe cycling habits [22]: all of these elements are closely related to the injury rates suffered by this population [23].

### Age as a modulator of road risk: A behavioral perspective

During the last few years, the evidence has systematically shown how behavioral trends (e.g., frequently observed road misbehaviors and protective factors) can be largely linked to the age of road users. On one hand, several age-based empirical researches have found that younger cyclists are more prone to perform deliberate risky cycling misbehaviors than older ones, thus increasing their likelihood to get involved in a cycling crash [24,25]. Furthermore, in a recent study, Mwakalonge, White & Siuhi [26] found that, compared to adult and aging cyclists (especially >50 years old), younger riders suffer more crashes related to the use of distracting elements such as the cellphones and other portable electronic devices. However, the evidence supports that, although older cyclists trendy have a higher road risk perception than younger ones [27], they could be more likely to be involved in traffic crashes: this is a consequence of physiological changes experienced with age, such as decreased balance, vision range, hearing and reaction time [25,28]. Another relevant study performed on the subject is the one by Ma et al. [29], in which age proved to have a significant impact on risky behaviors and accidents suffered by bike users.

Furthermore, it is important to highlight that aging cyclists are more likely to suffer severe injuries derived from cycling crashes, especially when the law and road culture do not strengthen the use of passive safety elements (PSEs) such as helmets. In this regard, several physiological vulnerability factors, such as a lower bone density, summed to a high prevalence of osteoporosis, may explain a higher rate of bone fracture, hospitalization and mortalities among elderly people involved in a traffic crash [30]. Finally, official data retrieved from different countries show that, linked to the *boom* of urban bicycling, injury rates among aging cyclists (admitted to hospitals after suffering a cycling crash) have been increasing considerably during the last few years, with head injuries being the most common cause of fatalities among them [4,21,31].

### Objectives and hypotheses

Thus, the objectives of this study focused on some fundamental comparisons: first, we compared individual factors modulating bicycling safety such as risky and positive behaviors, distractions and risk perception, between aging adults and other age groups (young and adult) of cyclists; and second, we compared mental health indicators between cyclists belonging to different age groups.

Based on the evidences mentioned in the literature review, this study had two hypotheses: first, regarding cycling risk-related factors, we expected to find higher rates of risky behaviors (errors and violations while riding), distractions and cycling crashes in cyclists younger than 50 (groups of young and adult cyclists), and more elements that decrease traffic crash risk, such as protective behaviors, risk perception and a higher knowledge of traffic norms, in adults over 50.

Second, and considering the benefits of cycling for mental health, especially in the aging population (e.g., reduced stress and increased subjective well-being), it was hypothesized that older adults may report lower indexes of psychological distress and higher rates of life satisfaction.

## Methods

### Sample

This study analyzed the data retrieved from a full sample of 911 respondents (39% females and 61% males) from two Latin American countries that have experienced a substantial growth of urban cycling during the last years: Colombia (*n* = 691) and Argentina (*n* = 220). The sample was divided in three age-based segments: a first (reference group) of *n* = 147 aging adults (over 50; *M* = 57.41 years), a second group of *n* = 385 adults (26–50; *M* = 32.80 years), and a third group of *n* = 379 young adults (25 or younger; *M* = 21.70 years). Demographic features and key cycling habits of the sample are presented in Table 1.

**Table 1:** Demographic data, cycling patterns and cycling crash rates of the sample.

**Feature**	**Category**	**Frequency**	**Percentage**
Gender	Female	347	39%
	Male	564	61%
Educational level	Primary studies or lower	1	.1%
	Secondary-high school	101	11.1%
	Technical studies	82	9%
	University studies	402	71.8%
	Other	257	28.2%
**Feature**	**Group**	**Mean**	**SD**
Age	Young	21.7	2.2
	Adult	32.8	6.1
	Aging Adults	57.4	6.1
Cycling time per week (hours)	Young	8.1	7.4
	Adult	6.5	5.6
	Aging Adults	4.6	4.0
Mean length of most frequent trips (minutes)	Young	42	28.1
	Adult	44	32.7
	Aging Adults	46	21.5
Cycling crashes suffered (last 5 years)	Young	.9	1.2
	Adult	.6	.9
	Aging Adults	.4	.8

### Study design and procedure

For this cross-sectional study, we used a convenience sampling technique. Convenience sampling constitutes one of the most employed non-probabilistic methods, and it is based on the accessibility to the study population. This sampling method was chosen considering that it is quick, inexpensive and flexible, and that it can be easily adapted to the time availability of participants [32]. For this purpose, individuals that were previously included in a collaborative database used by universities and research groups for research purposes were invited to take part in the study. This task was carried out in the two aforementioned Latin American countries (Colombia and Argentina), in which our cooperative research staffs and organizations had coverage and where previous cooperative research experiences had already taken place. As for the procedure, participants were invited to take part in the research by means of an electronic form sent via e-mail: in other words, by sending an electronic invitation for each potential participant. It is important to remark that the study was quite rigorous, guaranteeing the anonymity of participants and emphasizing the fact that data would be used for research purposes only, as required by the Ethics Committee (see also section *Ethics*). Contributors (respondents) did not perceive any material/economic reward for their participation in the study. This was stated during the presentation of the e-questionnaire, that also required the reading and acceptance of an informed consent statement prior to answering the questions; participants were invited to confirm their agreement with the study aims and procedures in a special box contained in the form. E-forms were fully completed during a period of approximately 7 months by a total of *n* = 911 cyclists, and the response rate was around 60%, with a number of approximately 1,500 sent invitations.

### Description of the questionnaire

The questionnaire was structured in three sections. The first part asked about individual and demographic variables, such as age, gender, educational level and cycling-related aspects, such as hours spent riding per week, mean length of the most frequent cycling trips, and cycling crashes (regardless of their severity) suffered during the previous five years.

As for the second part, self-reported risky cycling behaviors were assessed using the validated version of Useche’s Cyclist Behavior Questionnaire (CBQ) [33], a self-report measure of the high-risk (errors and violations) and positive riding behaviors of cyclists. This Likert scale uses a frequency-based response scale displaying 5 levels, and it is composed of 29 items distributed in three factors: *Violations* (*α =* .790), consisting of 8 items; *Errors* (*α =* .820), composed of 15 items; and *Positive Behaviors* (*α =* .740), consisting of 6 items. A fourth factor, *Risky Behaviors* (*α =* .895), can be built up through the sum of the 23 items contained in *Errors* and *Violations*. Additionally, this scale includes a supplementary set of three short scales aimed at assessing a) risk perception, and b) knowledge of general traffic norms of riders applicable to different countries (Cyclist Risk Perception and Regulation Scale; RPRS). This is a Likert scale composed of 12 items, in which the degree of the risk perceived (7 items; *α* = .651) in objectively risky factors and the knowledge of the general road regulations (5 items; *α* = .719) are assessed. Finally, the questionnaire on cycling distractions is a dichotomous scale (8 items; *α* = .603) used for assessing the impact of potential distracting sources commonly experienced by participants [34]. The contents of the questionnaires included in this section of the instrument are fully available in S1 Appendix.

The third part of the survey addressed two indicators related with mental health: first, we used Goldberg’s General Health Questionnaire (GHQ-12) [35]. This short 12-item Likert scale aims at assessing different potential symptoms that may have affected the subject’s mental health in the form of psychological distress in a time lapse of one month. Secondly, we applied the Satisfaction with Life Scale (SWLS) [36], that consists of a short 5-item Likert tool for measuring global cognitive assesments of life satisfaction [36,37].

### Ethics

Once the authors considered all the aspects related to the ethics, procedures and data privacy of the project and submitted both the informed consent form and the research questionnaire for evaluation -mandatory step for applied studies involving human subjects-, the *Human Research Ethics Committee* of the University of Valencia gave its approval to the study (IRB approval number H1517828884105), which was framed within the macro-project entitled *Habits*, *Behaviors and Road Safety of Cyclists*.

### Statistical analysis (data processing)

First of all, we carried out the data curation, in order to enhance the basic aspects of the data that were to be analyzed. Once the data was clean and properly labelled, basic descriptive analyses on the study sample were performed in order to characterize the participants of the study according to their demographic features and cycling-related patterns. Furthermore, the instruments used in the study (i.e., Cyclist Behavior Questionnaire or CBQ, RPRS, GHQ and SWLS) were scored according to their own guidelines, and Pearson’ (bivariate) correlational analyses were employed to compare the measures of association between all the variables calculated. Considering the lack of proportionality between the sample sizes and variances of the sample sub-groups, confirmed via Levene’s test, age-based comparisons were performed through Brown-Forshyte’s (BF) robust mean tests. The performance of these analyses is suggested when: *a)* Fisher’s F test for ANOVA is not valid due to the lack of normality and homoscedasticity, and/or *b)* sub-sample sizes are quite unequal or disproportional due to characteristics of the sample, but the researcher seeks for an acceptable statistical power in alternative tests [38]. Specifically, BF test uses a different denominator for the equation of F in ANOVA and, instead of dividing by the mean square of the error, it is adjusted by using the variances observed in each age group. The interpretation of BF’s p-values is identical to the case of conventional ANOVA analyses. Finally, Post-Hoc tests (Tukey HSD) with 95% confidence intervals [CI] were performed in order to determine the significant differences between specific pairs of age groups. All statistical analyses were performed using ©IBM SPSS (Statistical Package for Social Sciences), version 24.0.

## Results

### Correlation analysis

In order to test the associations between study variables, Pearson’ bivariate correlation analyses were carried out. As a result, interesting significant associations were found between pairs of variables.

The age of cyclists was positively associated with other variables that contribute to decreasing the risk of suffering a cycling crash: positive cycling behaviors (*r* = .189**), knowledge of traffic norms (*r* = .365**), and risk perception (*r* = .240**). However, age was also found to be positively associated with cycling distractions (*r* = .167**). On the other hand, negative associations were found between age and riding errors and violations (risky road behaviors) (*r* = -.155* and *r* = -.295**, respectively), as well as with the amount of traffic crashes suffered while cycling during the last five years (*r* = -.190**). Apart from demographic and behavioral issues, risky road behaviors were negatively correlated to positive behaviors (*r* = -.348** for errors and *r =* -.445** for violations), knowledge of cycling traffic norms (*r* = -.269** for errors and *r =* -.158** for violations) and risk perception (*r* = -.175** for errors and *r =* -.219** for violations). Furthermore, cycling distractions were positively associated with errors (*r* = 187**), but not with deliberated violations of traffic norms.

As for the bivariate correlations existing between mental health indicators (psychological distress and life satisfaction), it was found that psychological distress is positively associated with both cycling errors (*r* = .206**) and violations (*r* = .129**), and negatively linked to positive behaviors (*r =* -.150**), traffic rule knowledge (*r =* -.302**) and risk perception (*r =* -.164**). Finally, the life satisfaction of cyclists was found to be positively associated with positive behaviors (*r =* 173**), rule knowledge (*r =* .271**) and a higher risk perception (*r =* .196**), but negatively related to risky behaviors (*r* = -.131** for errors and *r =* -.106** for violations) and cycling crashes (*r =* -.082**). This is, cyclists reporting a lower satisfaction with life also tend to perform more risky riding behaviors and to suffer more cycling crashes. The full set of correlations is available in Table 2.

**Table 2:** Bivariate correlations among cycling-related variables (full sample and sub-samples).

		**2**	**3**	**4**	**5**	**6**	**7**	**8**	**9**	**10**
**1**	Age (years)	-.257**	.217**	-.155**	-.295**	.189**	.365**	.240**	.167**	-.190**
**2**	Psychological Distress		-.635**	.206**	.129**	-.150**	-.302**	-.164**	.062	.063
**3**	Life Satisfaction			-.131**	-.106**	.173**	.271**	.196**	-.037	-.082*
**4**	Errors				.467**	-.348**	-.269**	-.175**	.187**	.228**
**5**	Violations					-.445**	-.158**	-.219**	.018	.355**
**6**	Positive Behaviors						.269**	.370**	-.006	-.188**
**7**	Knowledge of Traffic Rules							.336**	-.017	-.080*
**8**	Risk Perception								.057	-.048
**9**	Distractions while Riding									-.033
**10**	Cycling Crashes (5 years)									

Notes:

** Correlation is significant at 0.01 level (2-tailed);

* Correlation is significant at 0.05 level (2-tailed).

### Age-based comparative analyses: Mental health and risk factors

In order to compare the mental health and cycling-related indicators of aging adults with the ones of other age-based groups of cyclists, robust mean analyses (Brown-Forshyte tests) were carried out, revealing interesting differences. First of all, test results confirmed that age-based significant differences exist among the three groups (young people, adults, aging adults) for both mental health indicators considered in the study, i.e., psychological distress and satisfaction with life. In the case of psychological distress, the highest mean value was found in young cyclists (<25 years; *M =* 24.45; SD = 5.35) while the lowest average score corresponded to aging adults (*M =* 21.05; SD = 4.41).

On the other hand, the age-based group reporting the highest mean value for life satisfaction was the aging adults one (*M* = 27.97; SD = 5.36), while the lowest average of life satisfaction was reported by the other two groups of participants, both scoring almost identical means and with similar dispersion measures. The full set of descriptive data obtained for the different study variables is presented in Table 3.

**Table 3:** Descriptive data for study variables and age-based robust mean comparisons.

Study Variable	Age Group	Mean	SD^1^	SE^2^	95% CI^3^		Brown-Forshyte test			
					Lower	Upper	Statistic^4^	df1	df2	Sig.^5^
Psychological Distress	Young people	24.45	5.35	.28	23.90	25.00	25.97	2	731.07	< .001
	Adults	22.90	5.26	.27	22.37	23.44				
	Aging Adults	21.05	4.41	.37	20.33	21.77				
Life Satisfaction	Young people	24.69	6.39	.34	24.03	25.36	17.44	2	726.05	< .001
	Adults	24.70	6.66	.35	24.01	25.40				
	Aging Adults	27.97	5.36	.45	27.09	28.85				
Errors	Young people	9.01	5.89	.30	8.42	9.61	11.99	2	651.66	< .001
	Adults	7.27	6.22	.32	6.64	7.89				
	Aging Adults	6.68	5.71	.47	5.75	7.61				
Violations	Young people	6.63	4.15	.21	6.21	7.05	39.36	2	829.43	< .001
	Adults	5.49	4.12	.21	5.07	5.90				
	Aging Adults	3.46	2.99	.25	2.98	3.95				
Positive Behaviors	Young people	18.02	3.54	.18	17.66	18.38	13.38	2	620.50	< .001
	Adults	18.72	4.00	.20	18.32	19.12				
	Aging Adults	19.88	3.70	.31	19.28	20.49				
Knowledge of Traffic Rules	Young people	2.84	.73	.04	2.77	2.91	68.04	2	841.61	< .001
	Adults	3.05	.69	.03	2.98	3.12				
	Aging Adults	3.55	.49	.04	3.47	3.63				
Risk Perception	Young people	3.32	.49	.03	3.27	3.37	24.59	2	712.41	< .001
	Adults	3.45	.52	.03	3.39	3.50				
	Aging Adults	3.65	.44	.04	3.58	3.72				
Distractions while Riding	Young people	4.67	1.60	.08	4.51	4.83	8.29	2	529.41	< .001
	Adults	4.85	1.89	.10	4.66	5.04				
	Aging Adults	5.39	1.94	.16	5.08	5.71				
Cycling Crashes (5 years)	Young people	.89	1.18	.06	.77	1.01	18.322	2	746.79	< .001
	Adults	.59	.88	.05	.50	.68				
	Aging Adults	.38	.82	.07	.25	.51				

Notes:

^1^Standard Deviation;

^2^Standard Error;

^3^Interval at 95% of Confidence;

^4^Asymptotically F distributed;

^5^p-value for Robust Tests of Equality of Means.

As for cycling-related variables, age-based comparisons showed that risky road behaviors (errors and deliberated violations of traffic norms) present a similar trend among the three groups of cyclists: young riders reported the highest average of both errors and violations, and aging adults the lowest. In turn, aging adults were the ones reporting most positive (or protective) cycling behaviors (*M* = 19.88; SD = 3.70), and the highest values for rule knowledge (*M* = 3.55; SD = .49), risk perception (*M* = 3.65; SD = .44), and cycling crashes suffered along the last five years (*M* = .38; SD = .82). However, it is worth mentioning that, unlike what was initially expected, aging adults also have the highest rate of distractions while cycling (*M* = 5.39; SD = 1.94), in comparison with both groups of adult (*M* = 4.85; SD = 1.89), and young (*M* = 4.67 SD = 1.60) participants of the study.

### Post-Hoc comparisons

With the aim of determining specific differences in the scores obtained by the different study variables between pairs of age groups (procedure that conventional tests for comparing means such as One-way ANOVA, Student’s t, Welch and Brown-Forshyte do not allow for), Post-Hoc analyses with a confidence interval of 95% were performed, considering aging adults as reference group. Regarding the two mental health indicators used in this study, it was found that the psychological distress index of aging cyclists was significantly lower when compared with both groups of adults (*M*_dif_ = -1.85*) and young riders (*M*_dif_ = -3.40*). On the other hand, it was found that the life satisfaction of aging adults was significantly higher than the one reported by young (*M*_dif_ = 3.27*) and adult cyclists (*M*_dif_ = 3.26*). The results of Post-Hoc (Tukey HSD) tests and the details of the obtained confidence intervals are presented in Table 4.

**Table 4:** HSD (Tukey) Post-Hoc tests for comparing age-based scores between pairs of groups.

Dependent Variable	Group (I)^1^	Group (J)^2^	Diff. (I-J)^3^	SE^4^	Sig.^5^	95% CI^6^	
						Lower	Upper
Psychological Distress	Young people	Young people	1.54*	.38	< .001	.65	2.44
		Aging Adults	3.40*	.51	< .001	2.21	4.59
	Adults	Young people	-1.54*	.38	< .001	-2.44	-.65
		Aging Adults	1.85*	.50	< .01	.67	3.04
	Aging Adults	Young people	-3.40*	.51	< .001	-4.59	-2.21
		Adults	-1.85*	.50	< .01	-3.04	-.67
Life Satisfaction	Young people	Adults	-0.01	.48	N/S	-1.13	1.10
		Aging Adults	-3.27*	.63	< .001	-4.75	-1.81
	Adults	Young people	0.01	.48	N/S	-1.10	1.13
		Aging Adults	-3.26*	.63	< .001	-4.74	-1.80
	Aging Adults	Young people	3.27*	.63	< .001	1.81	4.75
		Adults	3.26*	.63	< .001	1.80	4.74
Errors	Young people	Adults	1.74*	.43	< .001	.72	2.76
		Aging Adults	2.33*	.58	< .001	.96	3.70
	Adults	Young people	-1.74*	.43	< .001	-2.76	-.72
		Aging Adults	.58	.58	N/S	-.78	1.95
	Aging Adults	Young people	-2.33*	.58	< .001	-3.70	-.96
		Adults	-.58	.58	N/S	-1.95	.78
Violations	Young people	Adults	1.14*	.29	< .001	.47	1.82
		Aging Adults	3.16*	.39	< .001	2.26	4.07
	Adults	Young people	-1.14*	.29	< .001	-1.82	-.47
		Aging Adults	2.02*	.39	< .001	1.12	2.93
	Aging Adults	Young people	-3.16*	.39	< .001	-4.07	-2.26
		Adults	-2.02*	.39	< .001	-2.93	-1.12
Positive Behaviors	Young people	Adults	-.70*	.27	< .05	-1.34	-.06
		Aging Adults	-1.86*	.37	< .001	-2.72	-1.00
	Adults	Young people	.70*	.27	< .05	.06	1.34
		Aging Adults	-1.16*	.36	< .01	-2.02	-.31
	Aging Adults	Young people	1.86*	.37	< .001	1.00	2.72
		Adults	1.16*	.36	< .01	.31	2.02
Knowledge of Traffic Rules	Young people	Adults	-.21*	.05	< .001	-.33	-.10
		Aging Adults	-.70*	.07	< .001	-.86	-.56
	Adults	Young people	.21*	.05	< .001	.10	.33
		Aging Adults	-.49*	.07	< .001	-.65	-.34
	Aging Adults	Young people	.70*	.07	< .001	.56	.86
		Adults	.49*	.07	< .001	.34	.65
Risk Perception	Young people	Adults	-.12*	.04	< .01	-.21	-.04
		Aging Adults	-.32*	.05	< .001	-.44	-.21
	Adults	Young people	.12*	.04	< .01	.04	.21
		Aging Adults	-.20*	.05	< .001	-.31	-.09
	Aging Adults	Young people	.32*	.05	< .001	.21	.44
		Adults	.20*	.05	< .001	.09	.31
Distractions while Riding	Young people	Adults	-.18	.13	N/S	-.48	.12
		Aging Adults	-.72*	.17	< .001	-1.13	-.32
	Adults	Young people	.18	.13	N/S	-.12	.48
		Aging Adults	-.54*	.17	0.005	-.95	-.14
	Aging Adults	Young people	.72*	.17	< .001	.32	1.13
		Adults	.54*	.17	0.005	.14	.95
Cycling Crashes (last 5 years)	Young people	Adults	.30*	.07	< .001	.13	.48
		Aging Adults	.51*	.10	< .001	.28	.74
	Adults	Young people	-.30*	.07	< .001	-.48	-.13
		Aging Adults	.21	.10	N/S	-.02	.44
	Aging Adults	Young people	-.51*	.10	< .001	-.74	-.28
		Adults	-.21	.10	N/S	-.44	.02

Notes:

^1^Reference Group;

^2^Contrasting Group;

^3^Mean Difference;

^4^Standard Error;

^5^*p*-value;

^6^Interval at 95% of Confidence;

*Significant at the level *p*<0.05.

As for road risk-related variables, Post-Hoc analyses allowed us to determine that aging adults commit significantly fewer riding errors and traffic violations than adult (*M*_dif_ = -.58*_errors_; *M*_dif_ = -.202*_violations_) and young cyclists (*M*_dif_ = -2.33*_errors_; *M*_dif_ = -3.16*_violations_). On the other hand, aging cyclists also perform positive behaviors more frequently than adult (*M*_dif_ = 1.16*) and young riders (*M*_dif_ = 1.86*). Furthermore, the knowledge of cycling traffic norms and road risk perception were significantly higher among cyclists over 50 years old, when compared to adult (*M*_dif_ = .49*_rule knowledge_; *M*_dif_ = .20*_risk perception_) and young cyclists (*M*_dif_ = .70*_rule knowledge_; *M*_dif_ = .32*_risk perception_). However, cycling distractions have been shown to affect aging cyclists more than the other two (*M*_dif_ = .54*_adult_; *M*_dif_ = .72*_young_) age groups of riders.

Finally, Post-Hoc analyses allowed us to determine that the rate of cycling crashes suffered by aging adults during the last five years were significantly less numerous when compared with the group of young cyclists (*M*_dif_ = -.51*), but not with adults aged between 26–50 years old (*M*_dif_ = -.21^N/S^).

**Fig 1:**
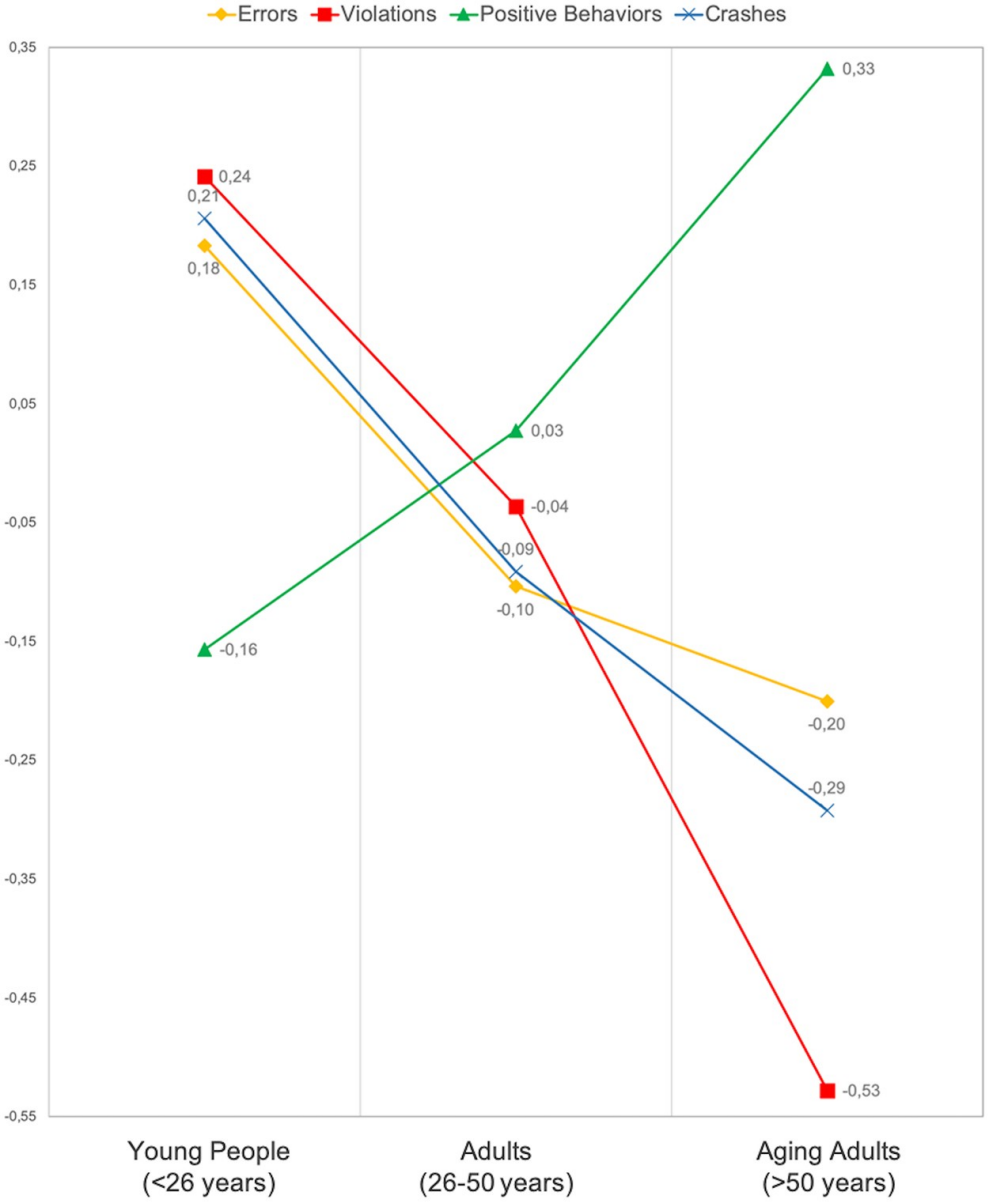
Graphical trends in risky behaviors -errors and traffic violations-, positive behaviors and traffic crash rates among cyclists in different age groups (standardized values).

Fig 1 graphically shows the mean (standardized) scores obtained in each subscale for the three factors of the CBQ (errors, traffic violations and positive behaviors) and the average number of cycling crashes suffered during the previous five years. It is striking to see how the group of younger cyclists (under 26 years) reports not only higher means of errors, violations and crashes, but also a considerably lower score in protective cycling behaviors. On the other hand, aging adults show lower means in the two variables related to risky cycling behavior (errors and violations), a relatively lower rate of cycling crashes and a higher score in positive behaviors performed while riding.

## Discussion and conclusion

The objective of this study was to analyze cycling safety-related factors and mental health indicators of aging cyclists in comparison with other age groups (young and adult cyclists). Overall, this study showed how aging adults from two countries of Latin America tend to report a more favorable state-of-affairs in cycling safety than younger ones. In other words, they perform less risky behaviors (unintentional errors and deliberated traffic violations), more protective behaviors (including the avoidance of cycling under adverse weather conditions and the frequent use of helmets and other protective features), and they have a higher risk perception and knowledge of cycling norms. However, the specific indicators related to each one of the three age groups should be nuanced and discussed.

### Young, adult and aging adult: Towards a “safer” kind of cyclist?

First of all, in the present study *young cyclists* presented a major latent risk, based on both their behavioral patterns and their self-reported cycling crashes suffered during the past few years (*M* = .89). In accordance, similar researches have described how age decreases behavioral risks among cyclists [39]; also, other studies involving other types of road users, especially motor-vehicle drivers, have found hazardous trends in terms of risky road behaviors [40–42], low risk perception [43,44] and a scarce knowledge of traffic norms [45–47] among young people, especially those under 25. In this regard, most studies dealing with young drivers have remarked that young road users constitute a crucial focus to address in road safety interventions [48].

Secondly, it is worth highlighting some key adverse outcomes obtained by *adult cyclists*: although their cycling crash rate is considerably lower (*M* = .59) than the one reported by young riders (*M*_dif_ = -.30*), it is, at the same time, significantly higher when compared to the crash rate of aging cyclists (*M*_dif_ = .21*). In this regard, different empirical studies performed during the last 35 years have shown that, while it is true that adult cyclists do not comparatively constitute a primary focus for cycling injuries and fatalities, their risk level is still high, and this implies a considerably heavy burden for healthcare systems and community settings [49–50]; also, more actions and policing for reducing behavioral risks and environmental factors affecting this group’s cycling safety are definitely required [51].

Thirdly, as we have mentioned above, *aging adults* seem to be, on one hand, the age segment of cyclists that: *a*) suffers the lowest rate of cycling accidents, significantly lower than the one reported by young cyclists (*M*_dif_ = -2.33*) and lower than the one corresponding to adult riders (*M*_dif_ = -.21^N/S^); *b*) compared to the other age groups, cyclists over 50 years old tend to perform less risky behaviors, engaging in more protective ones instead, and *c*) present a *better* result in both mental health indicators that we considered, meaning psychological distress (lowest) and life satisfaction (highest). As for the last one, the findings of this study -based on significant correlations- have linked mental health indicators and traffic safety. Different studies, such as the ones performed by Abdoli et al. [52] and McDonald, Sommers & Fargo [53] have already found significant associations between the mental health of drivers and their risky behaviors on the road.

In other words, the findings of this research -if we mainly, but not exclusively focus on risky and protective behaviors- support the idea that road safety figures of aging adults using bicycles are comparatively *better* than the ones of other age groups; but, at the same time, traffic crashes involving them could be even less prevalent if we took care of another factor affecting the safe performance of cyclists: distractions on the road.

### Distracted cycling and elderly users: A challenge to overcome

During their trips, both cyclists and other road users are commonly exposed to a great amount of information, traditionally translated into the demands of different factors such as traffic signaling, billboards, noise and the risky behaviors of other road users [29,34,54].

However, the systematic introduction of other elements such as electronic devices (e.g., cellphones, GPS navigators, earbuds) has substantially increased the likelihood of cyclists to get distracted and, subsequently, suffer traffic incidents that range from near-misses to fatal crashes [55,56]. In this sense, and although some recent studies have focused on the problem of young cyclists, the growing naturalization of e-devices in everyday life is also affecting transportation dynamics of cyclists belonging to all age segments [27,57,58]. Thus, it is worth discussing the role of distractions in traffic crashes involving bicycle riders. In a recent empirical study performed on cyclists from 20 different countries, Useche, Alonso, Montoro & Esteban [34] found that the mechanism through which distractions represent a threat for cycling safety is their relationship with cycling errors as a statistical mediator.

In other words, although such distractions are not causally linked to the crash, they precede unintentional risky behaviors that may result in cycling crashes. In fact, the hazardousness of cycling distractions caused by the use of electronic devices in urban biking has been compared to the use of cellphones among motor-vehicle drivers [53]. In addition to this, if we consider the specific case of aging cyclists, other factors such as diminished vision range, hearing and reaction times [28,29] may contribute to increasing the risk of causing or suffering serious accidents as a result of cycling distractions.

### Promoting a “safer & healthier” cycling for aging adults

At the beginning of this paper, we remarked how different benefits such as health improvements and environmental sustainability have been recognized as important predictors of urban cycling, and the positive outcomes of *active transportation* implying physical activity constitute the core reason for its promotion among the aging population. However, some key barriers and constraints still need to be addressed in most of Latin American countries [18,20]. Apart from the evident gaps in the cycling infrastructure, road safety education and bicycling culture of these countries [59,60], recent evidence has been found on the scarce policymaking aimed at protecting vulnerable groups of cyclists, in addition to the absence of legislation in the fields of training, use of passive safety elements and road safety education [22,61,62]. For instance, to the date there are no studies addressing neither the impact of road infrastructure not the current policymaking on aging adults’ road safety; furthermore, if the data give us the reason, this study constitutes the first behavioral approach using validated instruments to address the relationship between age, behavior and cycling safety in the context of Colombia and Argentina. However, more evidence is needed if we wish to develop effective and sustainable legislation aimed at protecting this age segment that, although suffering fewer accidents, is proportionally overrepresented in road accident figures [63].

In other words, beyond the positive health, social and environmental improvements that cycling may involve for both aging adults and younger cyclists [19,64], this and other empirical experiences applied to Latin America have remarked the undisputable role of not only promoting active transportation, but also enhancing the simultaneous development of policies and institutional strategies aimed at overcoming the risks of alternative transport means [65–67].

## Practical implications of the study

This research aimed to comparatively assess some key bicycling-related factors of aging adults in two Latin American countries, bearing in mind the outcomes of two other age groups: young (<26 years) and adult (26–50) cyclists. The results of this study suggest some relevant practical guidelines. In short, although the objective risk features may be relatively homogeneous, the age-based trends observed in the study variables allow for the identification of differential needs for each one of the groups, that should be considered for the design and performance of cycling safety-related interventions and policymaking. Concretely:

- Aging cyclists are less prone to perform risky cycling behaviors, and more likely to show protective ones while riding; they also present higher risk perception and rule knowledge, when compared to other age groups. However, they are more prone to suffer cycling distractions, and this fact could enhance their crash risk, which could be an interesting issue to address in road training tailored to aging cyclists.
- Although less prone to suffer cycling distractions, young cyclists (<26) were showed to be the age-group presenting the highest rates of risky behaviors, and the lowest rates of protective ones, together with risk perception and rule knowledge. Thus, interventions aimed at young cyclists should focus on these issues as a way of strengthening their cycling safety.
- Both this and other previous studies have emphasized the relevance of mental health in road safety. In this regard, we found how psychological distress and life satisfaction are correlated to cycling behavior. Although this study did not have a predictive value, the data allow for the consideration that mental health and well-being could have a relevant role in the road training of cyclists, focusing on risk avoidance and crash prevention.

## Limitations of the study and further research

This study followed a theoretical-based design, and used the data collected from a considerably large sample from two Latin American countries in which urban cycling has recently been growing. For this purpose, the data was carefully retrieved, cured and analyzed in consideration of the statistical parameters appropriate for each one of the performed analyses. However, this was a self-report-based study, and therefore it remains potentially vulnerable to some sources of bias that should be acknowledged. Firstly, it is important to emphasize the fact that self-report surveys and questionnaires have been related to common method biases (CMBs), that may elicit desirability among the participants, especially when they are asked about social habits and behaviors (i.e., *how do they behave on the road*?). In this regard, our questionnaire was emphatic on the following facts: *a)* its total anonymity; *b)* the absence of good/wrong answers, thus encouraging participants to provide frank answers with real value for the study, and *c)* the purpose of retrieving the data, which was merely scientific. Secondly, we would have liked to make further comparisons between both countries that, although quite similar for what concerns the situation of cyclists, may present certain differences that are worth investigating; however, the disproportionality of our sample sizes did not allow us to accomplish this task. Finally, we would like to remark the potential benefit of performing predictive models with bigger sample sizes for testing the potentially explanatory role of key variables such as age and mental health indicators in cycling crashes.

## Supporting information

S1 Appendix Questionnaires on cycling-related issues are fully available in this file. (DOCX)

S1 Dataset Raw data is available in the file (database) attached to the electronic version of this manuscript. (ZIP)
