# Supplementary material for: Well-being, behavioral patterns and cycling crashes of different age groups in Latin America: Are aging adults the safest cyclists?
Source: PLoS One. 2019 Aug 28;14(8):e0221864. doi: 10.1371/journal.pone.0221864 (PMC6713343; doi:10.1371/journal.pone.0221864)
Supplement: S1 Appendix — (DOCX) [file pone.0221864.s001.docx]

**S1 Appendix. Research Questionnaires**

**I. Cycling Behavior Questionnaire - CBQ; 29-item validated version** (Cite as [28])

Please estimate how often you do the following when cycling, using this scale:

*0 = Never; 1 = Almost never; 2 = Sometimes; 3 = Frequently; 4 = Almost always / always*

| **Item content** | **Frequency** | | | | |
| --- | --- | --- | --- | --- | --- |
| Cycling under the influence of alcohol and / or other drugs or hallucinogens. | 0 | 1 | 2 | 3 | 4 |
| Circulating against the traffic (wrong way). | 0 | 1 | 2 | 3 | 4 |
| Zigzagging between vehicles when using a mixed lane. | 0 | 1 | 2 | 3 | 4 |
| Handling potentially obstructive objects while riding a bicycle (food, packs, cigarettes ...). | 0 | 1 | 2 | 3 | 4 |
| Feeling that sometimes I'm going at a higher speed than I should be going at. | 0 | 1 | 2 | 3 | 4 |
| Crossing what appears to be a clear crossing, even if the traffic light is red. | 0 | 1 | 2 | 3 | 4 |
| Carrying a passenger on my bicycle without it being adapted for such a purpose. | 0 | 1 | 2 | 3 | 4 |
| Having a dispute in speed or "race" with another cyclist or driver. | 0 | 1 | 2 | 3 | 4 |
| Unintentionally crossing the street without looking properly, thus making another vehicle brake to avoid a crash. | 0 | 1 | 2 | 3 | 4 |
| Colliding (or being close to it) with a pedestrian or another cyclist while cycling distractedly. | 0 | 1 | 2 | 3 | 4 |
| Braking suddenly and being close to causing an accident. | 0 | 1 | 2 | 3 | 4 |
| Failing to notice the presence of pedestrians crossing when turning. | 0 | 1 | 2 | 3 | 4 |
| Not braking on a ‘‘Stop” or ‘‘Yield” sign and being close to colliding with another vehicle or pedestrian. | 0 | 1 | 2 | 3 | 4 |
| Braking very abruptly on a slippery surface. | 0 | 1 | 2 | 3 | 4 |
| While I am distracted, I do not realize that a pedestrian intends to cross a crosswalk, and do therefore I do not stop to let him or her do so. | 0 | 1 | 2 | 3 | 4 |
| Not realizing that a parked vehicle intends to leave and consequently having to brake abruptly to avoid a collision. | 0 | 1 | 2 | 3 | 4 |
| When driving on the right side, not realizing that a passenger is getting out of a vehicle or bus, and thus being close to hitting him or her. | 0 | 1 | 2 | 3 | 4 |
| Trying to overtake a vehicle that had previously used its indicators to signal that it was going to turn, consequently having to brake. | 0 | 1 | 2 | 3 | 4 |
| Misjudging a turn and hitting something on the road, or being close to losing balance (or falling). | 0 | 1 | 2 | 3 | 4 |
| Unintentionally, hitting a parked vehicle. | 0 | 1 | 2 | 3 | 4 |
| Failing to be aware of the road conditions and falling over a bump or hole. | 0 | 1 | 2 | 3 | 4 |
| Confusing one traffic signal with another, and maneuvering according to the latter. | 0 | 1 | 2 | 3 | 4 |
| Trying to brake but not being able to use the brakes properly due to poor hand positioning. | 0 | 1 | 2 | 3 | 4 |
| I stop and look at both sides before crossing a corner or intersection. | 0 | 1 | 2 | 3 | 4 |
| I try to move at a prudent speed to avoid sudden mishaps or braking. | 0 | 1 | 2 | 3 | 4 |
| I usually keep a safe distance from other cyclists or vehicles. | 0 | 1 | 2 | 3 | 4 |
| When I use the bike path (or bike-lane), I always use the indicated lane. | 0 | 1 | 2 | 3 | 4 |
| I avoid circulating under adverse weather conditions. | 0 | 1 | 2 | 3 | 4 |
| I avoid circulating if I feel very tired or sick. | 0 | 1 | 2 | 3 | 4 |

**II. Cyclist Risk Perception and Regulation Scale**

Please indicate your level of agreement with the following statements, regarding your cycling experience, using this scale:

**Risk Perception**

0 = Strongly disagree; 1 = Disagree; 2 = Neither agree nor disagree; 3 = Agree; 4 = Strongly agree

| I understand the potential consequences of being involved in a traffic accident, for example, with another vehicle | 0 | 1 | 2 | 3 | 4 |
| --- | --- | --- | --- | --- | --- |
| I perceive potentially higher risks for my integrity when I ride a bicycle, than when I am on board of a motorized vehicle | 0 | 1 | 2 | 3 | 4 |
| I am aware of the other vehicles that surround me on the road | 0 | 1 | 2 | 3 | 4 |
| I realize that there are signaling and infrastructure problems that can affect my safety | 0 | 1 | 2 | 3 | 4 |
| I believe that driving under the influence of certain substances (alcohol, illegal and / or prescribed drugs) affects my ability to ride well | 0 | 1 | 2 | 3 | 4 |
| I am aware of the risks involved in using headphones and cellphones while I ride the bicycle | 0 | 1 | 2 | 3 | 4 |
| Riding in urban areas is especially risky, considering the number of vehicles and the complexity of the roads | 0 | 1 | 2 | 3 | 4 |

**Self-reported Rule Knowledge**

0 = Strongly disagree; 1 = Disagree; 2 = Neither agree nor disagree; 3 = Agree; 4 = Strongly agree

| I readily recognize traffic signals | 0 | 1 | 2 | 3 | 4 |
| --- | --- | --- | --- | --- | --- |
| I know the basic rules governing other types of vehicles | 0 | 1 | 2 | 3 | 4 |
| I believe that pedestrians should always have the priority, even with respect to cyclists | 0 | 1 | 2 | 3 | 4 |
| I easily identify areas prohibited to traffic or bicycle parking | 0 | 1 | 2 | 3 | 4 |
| I know the bicycle safety regulations of my city | 0 | 1 | 2 | 3 | 4 |

**III. Cycling distractions** (Cite as [48])

Normally, do these factors distract you and/or impair your cycling performance during your journeys?

| **Potential distracting source** | **No** | **Yes** |
| --- | --- | --- |
| Text messages or chats | 0 | 1 |
| Phone calls | 0 | 1 |
| Billboards | 0 | 1 |
| People that I find attractive | 0 | 1 |
| My own thoughts or concerns | 0 | 1 |
| Weather conditions | 0 | 1 |
| The behavior of other users of the road | 0 | 1 |
| The obstacles in the way | 0 | 1 |
